# Testosterone Modulates Altered Prefrontal Control of Emotional Actions in Psychopathic Offenders^1^,^2^,^3^

**DOI:** 10.1523/ENEURO.0107-15.2016

**Published:** 2016-02-08

**Authors:** Inge Volman, Anna Katinka Louise von Borries, Berend Hendrik Bulten, Robbert Jan Verkes, Ivan Toni, Karin Roelofs

**Affiliations:** 1 Sobell Department of Motor Neuroscience and Movement Disorders, UCL Institute of Neurology, University College London, London WC1N 3BG, United Kingdom; 2 Behavioural Science Institute, Radboud University Nijmegen, 6525 HR, Nijmegen, The Netherlands; 3 Donders Institute for Brain, Cognition and Behavior, Radboud University Nijmegen, 6525 EN, Nijmegen, The Netherlands; 4 Department of Psychiatry, UMC Sint Radboud, 6525 GA, Nijmegen, The Netherlands; 5 Pompestichting, 6532 CN, Nijmegen, The Netherlands

**Keywords:** amygdala, connectivity, emotion, fMRI, prefrontal, psychopathy

## Abstract

Psychopathic individuals are notorious for their controlled goal-directed aggressive behavior. Yet, during social challenges, they often show uncontrolled emotional behavior. Healthy individuals can control their social emotional behavior through anterior prefrontal cortex (aPFC) downregulation of neural activity in the amygdala, with testosterone modulating aPFC–amygdala coupling. This study tests whether individual differences in this neuroendocrine system relate to the paradoxical lack of emotional control observed in human psychopathic offenders. Emotional control was operationalized with an fMRI-adapted approach–avoidance task requiring rule-driven control over rapid emotional responses. Fifteen psychopathic offenders and 19 matched healthy control subjects made approaching and avoiding movements in response to emotional faces. Control of social emotional behavior was required during affect-incongruent trials, when participants had to override affect-congruent, automatic action tendencies and select the opposite response. Psychopathic offenders showed less control-related aPFC activity and aPFC–amygdala coupling during trials requiring control of emotional actions, when compared with healthy control subjects. This pattern was particularly pronounced in psychopathic individuals with high endogenous testosterone levels. These findings suggest that reduced prefrontal coordination underlies reduced behavioral control in psychopathic offenders during emotionally provoking situations. Even though the modest sample size warrants replication, the modulatory role of endogenous testosterone on the aPFC–amygdala circuit suggests a neurobiological substrate of individual differences that is relevant for the advancement of treatment and the reduction of recidivism.

## Significance Statement

Psychopathic criminals are commonly seen as instrumentally abusive and emotionally callous, yet social challenges often trigger uncontrolled emotional behavior in those individuals. This study shows how this paradoxical aspect of psychopathy relates to altered neuroendocrine interactions between testosterone and the cerebral circuit coordinating emotional action tendencies. The anterior prefrontal cortex, a region necessary for controlling emotional behavior, showed blunted responses and reduced connectivity with the amygdala in psychopathic criminals engaged in controlling their emotional action tendencies. This cerebral pattern was strongest in psychopathic individuals with high endogenous testosterone levels. This neuroendocrine signature of altered emotional control highlights the relevance of considering the testosterone level of individual psychopathic patients during treatment of their impulsive behavior.

## Introduction

Psychopathy is a disorder often associated with blunted emotional responding and increased goal-directed behavior (Blair, 2010; Anderson and Kiehl, 2012). On the other hand, offenders with psychopathy also show a paradoxical increase in impulsive behavior and uncontrolled aggression after emotional provocations (Cornell et al., 1996; Hare, 2003; Patrick et al., 2005; Malterer et al., 2008; Blair, 2010; Anderson and Kiehl, 2012), which may be related to heightened testosterone levels (Stålenheim et al., 1998; Dolan et al., 2001). These two aspects of psychopathy are also distinguished within the most commonly used psychopathy checklist, the Psychopathy Check List-Revised (PCL-R), potentially reflecting differing traits among psychopathic individuals (Hare, 2003; Anderson and Kiehl, 2012). Importantly, enhanced difficulty in controlling emotional impulses, a crucial component of criminal psychopathy associated with PCL-R factor 2, has been largely neglected by cognitive neuroscience. Yet, the clinical relevance of this cognitive trait is large: reduced behavioral control and increased impulsivity predict recidivism in psychopathic offenders (Walters, 2003), and behavioral control in psychopathic offenders appears particularly fragile when dealing with emotionally relevant behavior (Hare, 2003; Blair et al., 2005, chapter 7; Malterer et al. 2008). Accordingly, understanding the neurobiological systems underlying the altered control of social emotional behavior in psychopathic individuals is relevant for improving currently available interventions, which are plagued by low treatment response and high recidivism (Hare, 2003). Here we study those neuroendocrine systems in a group of psychopathic offenders engaged in an experimental paradigm that requires rule-driven control of emotional behavior.

Previous investigations of psychopathy showed altered reactivity to emotional material in several brain regions that include the anterior part of the PFC (aPFC) and the amygdala (Anderson and Kiehl, 2012; Blair, 2013; Decety et al., 2015). Furthermore, individuals with psychopathy showed decreased functional and anatomical connectivity between the PFC and amygdala at rest (Craig et al., 2009; Motzkin et al., 2011), an indication that these brain regions might have a reduced ability to interact effectively. Studies in healthy participants have shown that this cerebral circuit is necessary for implementing the control of emotionally relevant actions (Volman et al., 2011a). Namely, aPFC downregulates neural processing in the amygdala during emotional control (Volman et al., 2011a, 2013), while high levels of endogenous testosterone reduce such control-related connectivity between aPFC and amygdala (Volman et al., 2011b). Those findings raise the possibility that aPFC–amygdala connectivity is altered when psychopathic offenders need to control emotionally relevant actions, with high levels of endogenous testosterone exacerbating that altered connectivity.

**Figure 1.**
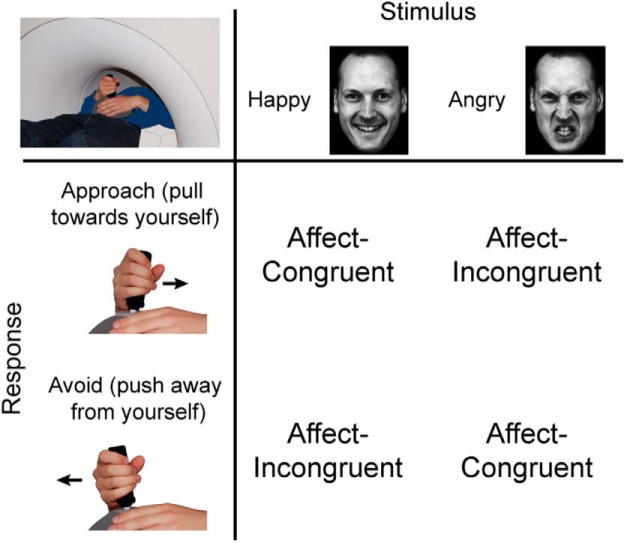
The emotional control AA task. The AA task involved the presentation of happy and angry faces, and the performance of approach and avoidance responses. During the AA task, the participants had to select their response according to the perceived emotion of the face. At the beginning of each block of 12 trials, the participants received instructions on whether to pull the joystick toward themselves (approach) or push it away (avoid) when seeing a face with a particular emotion. When viewing happy or angry faces, automatic stimulus–response tendencies trigger corresponding approach or avoidance actions. These tendencies could be followed during the affect-congruent condition (approach–happy, avoid–angry). In contrast, when task instructions required participants to avoid happy faces or to approach angry faces, automatic tendencies needed to be controlled and overridden with the instructed response (affect-incongruent condition). Participants saw the faces and moved the joystick while lying in a MR scanner (top left corner of the table). Figure adapted from Volman et al. (2011a, 2013).

This study tests these hypotheses by measuring brain activity with functional magnetic resonance imaging (fMRI) in 15 psychopathic criminals and 19 matched healthy control subjects dealing with a challenge to control their emotional behavior. The psychopathy sample was obtained by focused and comprehensive screening excluding confounds that are frequently associated with random criminal sampling (e.g., medication use, comorbidity). The social approach–avoidance (AA) task was used to provide reliable indexes of control over social emotional behavior (Fig. 1; Roelofs et al., 2009; Volman et al., 2011a,b). Behaviorally, psychopathic participants previously showed altered AA behavior to explicitly approaching and avoiding emotional faces (Von Borries et al., 2012). Similar findings occurred after testosterone administration in healthy participants (Enter et al., 2014). Interestingly, a more subtle version of the AA task has been shown to be sensitive to testosterone-related alterations and genetic variations in the aPFC–amygdala pathway, while keeping behavior constant across experimental groups (Volman et al., 2011b, 2013), opening the way for isolating neural vulnerability factors (Price and Friston, 1999) in psychopathy. During this task, participants respond to affective faces (happy, angry) presented for a short time with approach and avoidance movements. Automatic emotional tendencies (approach–happy and avoid–angry faces; affect-congruent response conditions) need to be controlled during affect-incongruent response conditions in order to apply the counterintuitive action of approaching angry and avoiding happy faces (Chen and Bargh, 1999; Roelofs et al., 2009). Healthy participants respond more slowly and rely more strongly on the aPFC when emotional control is required, operationalized by the differences evoked between affect-incongruent and affect-congruent trials (Roelofs et al., 2009; Volman et al., 2011b). Accordingly, this study tests whether exerting control over emotionally relevant actions is reflected by reduced functionality of the aPFC–amygdala circuit in psychopathic individuals, suggesting less prefrontal regulation of emotional actions. In addition, it sets out to test whether this alteration is intensified by high levels of endogenous testosterone.

## Materials and Methods

### Participants

The psychopathic group was recruited from in-patient populations of the Pompestichting and Oldenkotte, forensic psychiatric institutes (TBS-clinics) in the Netherlands. TBS-clinics are facilities for criminal offenders with a mental disorder treated on behalf of the state.

Seventeen male psychopathic violent offenders (age range, 23-56 years) participated; all had received a diagnosis with a PCL-R score of ≥26, according to European standards (Hare et al., 1991; Rasmussen et al., 1999; Hildebrand et al., 2004). PCL-R consensus scores were obtained by trained clinicians based on a structured PCL-R interview, clinical status, and history. After the independent scoring, the two raters compared their scores and came to the consensus score. When no consensus could be found, a third independent rater was included in the process. Dutch versions of the National Adult Reading Test and Edinburgh Handedness Inventory were used to assess IQ levels and right-handedness (Oldfield, 1971; Schmand et al., 1991). Twenty-one healthy male control subjects (HCs) matched for age, right-handedness, and IQ, without criminal records or history of psychiatric disorders, were recruited from staff of the clinics. All participants received oral and written information about the experiment and gave written informed consent according to guidelines of the local ethics committee (Commissie Mensengebonden Onderzoek region Arnhem-Nijmegen). Psychiatric exclusion criteria consisted of neurological, axis-I, and axis-II disorders, besides antisocial personality disorder for the psychopathic group. They were screened for these exclusion criteria by trained psychologists using Dutch versions of the Structured Clinical Interview (SCID; Groenestijn et al., 1999) and Mini-International Neuropsychiatric Interview (MINI; Van Vliet et al., 2000) for *Diagnostic and Statistical Manual of Mental Disorders*, 4th edition, disorders. All participants were asked about drug use and medical/neurological history to exclude the following: alcohol use of >3 units/day, cannabis, or other illicit drug use 1 week before, psychotropic medication other than oxazepam 5 d before, 1 unit of alcohol or oxazepam use within 24 h before the experiment; history of trauma capitis; visual and auditive disorder; and neurological disorder. Furthermore, general exclusion criteria for MRI experiments were applied. Two psychopathic patients (PPs) and two HCs were excluded from the analyses, due to incomplete scanning procedures (1 PP, 1 HC) or too many errors on the task (>16%, representing the outlier with a *z*-score >3). The final groups did not differ in age, IQ, and handedness (see Table 1).

**Table 1:** Demographical data

	Psychopathic offenders (*n* = 15)	HCs (*n* = 19)	*p* value
Age	37.8 (7.9)	40.7 (10.3)	0.368
IQ	101 (10)	102 (9)	0.761
Handedness	50.7 (81)	59.2 (62)	0.729
PCL-R total	30.4 (3.5)		
PCL-R F1	12.1 (2.6)		
PCL-R F2	14.1 (2.3)		

Values are presented as the mean (SD), unless otherwise indicated. F1, factor 1; F2, factor 2.

### Procedure

Two test sessions took place. During the first session, right-handedness, IQ, MINI, and SCID were assessed. During the second session, participants completed several questionnaires upon arrival in the laboratory, including the State-Trait Anxiety Inventory (STAI) to measure anxiety levels (Spielberger, 1983). Next, they provided saliva for the testosterone measurement. Afterward, participants were positioned in the 1.5 T MR scanner and familiarized with the task setup. Immediately after this, the fMRI session started with the AA task (duration, 30 min) followed by another task (not included in this report). After a short break outside the scanner, the anatomical scan (duration, 5 min) and an unrelated task were acquired in the side-by-side 3 T MR scanner.

### Experimental task

The AA task consisted of 24 blocks (with 12 trials per block and a baseline period of 21-24 s) during which participants had to respond to visually presented faces either by pulling a joystick toward themselves (approach) or by pushing it away from themselves (avoid; Fig. 1). The participants had to categorize faces as happy, angry, and neutral (filler items), based on their affective expressions. During each block, two of the three affective expressions were presented as stimuli, because only two responses could be given to categorize the stimulus. This resulted in six different block types each used four times, representing the affect (happy–angry, happy–neutral, angry–neutral) × movement (approach–avoid) combinations. At the start of each block, participants received written instructions regarding the required response mapping. The affect × movement combinations were pseudorandomly and evenly distributed (with no affect combination repetition), and the combination of the first block was counterbalanced across participants. Within each block, affective expressions and gender types were pseudorandomly presented, avoiding three or more sequential presentations of the same expression/gender, and two presentations of the same facial model. Each face was presented for 100 ms, preceded by a 300 ms blank screen, and followed by the participant’s response, a blank screen, and by a pseudorandom intertrial interval (ITI; 1-3 s). A baseline period of 21-24 s preceded each block. The faces were from 36 models (18 male) obtained from several databases (Ekman and Friesen, 1976; Matsumoto and Ekman, 1988; Lundqvist et al., 1998; Martinez and Benavente, 1998), each showing all expressions. The pictures were in grayscale, matched for brightness and contrast values, and displayed against a black background. To exclude influence from hair and nonfacial contours, the faces were trimmed. Joystick displacements of >80% along the sagittal plane within 2 s from stimulus presentation were marked as valid responses. Invalid responses were signaled for 1 s with written feedback stating “you did not move your joystick far enough.” After moving the joystick, participants had to return to the starting position (defined as the central area extending 20% along the sagittal plane) before the end of the ITI. Otherwise, visual feedback indicated “return the joystick to the starting position,” and the ITI was repeated after participants returned the joystick. The training at the beginning consisted of six blocks; one block of eight trials for each of the six affect × movement combinations. Different visual stimuli were used during the training and scanning blocks.

### Materials and apparatus

The fMR images were acquired on a 1.5 T MRI scanner (Avanto, Siemens Medical Systems) with an eight-channel head coil using a multiecho generalized autocalibrating partially parallel acquisitions (GRAPPA) sequence [Poser et al., 2006; repetition time (TR), 2.14 ms; five echo times (TEs), 9.4/21/33/44/56 ms; 34 transversal slices; ascending acquisition; distance factor, 17%; effective voxel size, 3.3 × 3.3 × 3.5 mm; field of view (FOV), 212 mm]. High-resolution anatomical images were acquired on a 3 T MRI scanner with a 32-channel head coil using a magnetization prepared rapid gradient echo sequence (TR, 2300 ms; TE, 3.03 ms; 192 sagittal slices; voxel size, 1.0 × 1.0 × 1.0 mm; FOV, 256 mm).

An MR-compatible joystick (Fiber Optic Joystick, Current Designs; sampling rate, 550 Hz) was placed on participants’ abdomens to ensure comfortable push-and-pull movements (Fig. 1). Participants wore MR-compatible headphones to reduce scanner noise (Commander XG MRI Audio System, Resonance Technologies). Stimuli were projected at the center of a screen, viewed via a mirror above the participant’s head, with a visual angle of 4° × 6° (width × height). Stimuli presentation and acquisition of joystick positions were controlled by a PC running Presentation version 13 (http://www.neurobs.com).

### Salivary measurements

Participants filled two Salicaps (IBL) with saliva for testosterone measurement, which were stored at −25°C. Testosterone concentration was measured using competitive chemiluminescence immunoassay with a sensitivity of 0.0025 ng/ml (IBL International, Tecan). Intra-assay and interassay coefficients are between 10% and 12%. To control variables influencing testosterone levels, participants were instructed to refrain from any food, cigarettes, and drinks (except water) for 1 h before the experiment.

### Behavioral analysis

Behavioral data was analyzed using MATLAB version 7.9 (MathWorks) and PASW Statistics 18 (SPSS Inc.). First, to obtain a precise measure of movement onset [reaction time (RT)], the joystick movement for each trial was reconstructed using the joystick displacement measurements. Excluded trials showed a joystick movement in the wrong direction, an extreme RT (<150 or >1500 ms), peak velocity (<0.1 cm/s), or movement time (>400 ms); or an error rate of above chance level in a block (in that case, the whole block was excluded). RTs and testosterone levels were log transformed to obtain a normal distribution. Second, following previous studies (Roelofs et al., 2009; Volman et al., 2011b), we conducted three-way repeated-measures ANOVA (ANCOVArm) on the mean RT and error rates, with factors group (PP, HC), movement (approach, avoid), and valence (happy, angry), including standardized testosterone and STAI state as covariate. A measure of anxiety (STAI) was included to account for the effects of psychopathy type (e.g., primary vs secondary); and the possible effects on emotional behavior, hormonal levels, amygdala, and prefrontal cortex functioning (Freitas-Ferrari et al., 2010; Koenigs et al., 2011; Giltay et al., 2012; Fouche et al., 2013). The α-level was set at *p* < 0.05.

### Functional MRI data

#### Single-subject analyses

Imaging data were preprocessed and analyzed using SPM8 (Statistical Parametric Mapping; http://www.fil.ion.ucl.ac.uk/spm). The first four volumes of each participant’s dataset were discarded to allow for *T*_1_ equilibration. Given the multiecho GRAPPA MR sequence (Poser et al., 2006), head motion parameters were estimated on MR images with the shortest TE (9.4 ms), since these are least affected by possible artifacts. These motion correction parameters, estimated using a least-squares approach with six rigid body transformation parameters (translations, rotations), were applied to the five echo images collected for each excitation. After spatial realignment, the five echo images were combined into a single MR volume using an optimized echo weighting method (Poser et al., 2006). The time series for each voxel was temporally realigned to the first slice in time. The *T*_1_-weighted image was spatially coregistered to the mean of the functional images. The fMRI time series were transformed and resampled at an isotropic voxel size of 2 mm into standard Montreal Neurological Institute (MNI) space by unified segmentation and normalization using the coregistered *T*_1_-weighted image (Ashburner and Friston, 2005). The normalized functional images were spatially smoothed using an isotropic 8 mm full-width at half-maximum Gaussian kernel.

The fMRI time series of each subject were further analyzed using an event-related approach in the context of general linear model, including the following effects: approach–happy, approach–neutral, approach–angry, avoid–happy, avoid–neutral, and avoid–angry. Trials excluded from behavioral analyses and periods of instructions or feedback were modeled as regressors. Vectors describing the time of picture presentation (onset) and RT of each event (duration) were convolved with the canonical hemodynamic response function. Potential confounding effects of residual head movement were modeled using original, squared, cubic, first-order, and second-order derivatives of the movement correction parameters (Lund et al., 2005). Three further regressors, describing the time course of signal intensities of white matter, CSF, and the portion of the MR image outside the skull were also added. This procedure accounts for image intensity shifts due to hand movements within or near the magnetic field of the scanner (Verhagen et al., 2006). Finally, fMRI time series were high-pass filtered (cutoff 120 s). Temporal autocorrelation was modeled as a first-order autoregressive process.

#### Group analyses

Consistent effects across participants and between groups were tested using a random-effects multiple regression analysis that included six contrast images (approach–happy, approach–neutral, approach–angry, avoid–happy, avoid–neutral, avoid–angry) per participant. Together, these images represented the estimated cerebral effects from 12 conditions of the experimental design [group (PP, HC) × valence (happy, neutral, angry) × response (approach, avoid)]. Standardized log-transformed testosterone and standardized STAI state levels were included in the multiple regression analysis as condition-specific [group (PP, HC) × valence (happy, neutral, angry) × response (approach, avoid)] regressors, generating another 12 regressors per variable.

All analyses assessed the congruency effect, reflecting task-related differences of affect-incongruent (approach–angry, avoid–happy) versus affect-congruent trials (approach–happy, avoid–angry; Roelofs et al., 2009; Volman et al., 2011b). We considered two effects. First, to test for general effects of congruency, we performed an analysis on the congruency effect over both groups and for each group separately. When assessing the effects of one group explicitly, we also tested whether those effects were specific to that group and were significantly weaker in the other group (at *p* < 0.05 uncorrected) by masking the statistical map describing the congruency effect in the first group (using multiple comparisons correction, see below) with the statistical map describing the group × congruency contrast. Second, to test whether testosterone differentially modulated the control of emotionally relevant actions in the groups, we performed a group × congruency contrast on the regressor parametrizing interindividual differences in testosterone on task-related conditions. If such an interaction is present, the testosterone modulation on the congruency effect of each group separately is considered. In addition to whole-brain analyses, we used a volume of interest (VOI) on coordinates previously found to be modulated by testosterone during the congruency effect in healthy students (two 8-mm-radius spheres centered on the following MNI coordinates: *x*, −30; *y*, 58; and z, 2; and *x*, 32; *y*, 54; and *z*, 8; Volman et al., 2011b).

The reported activations are corrected for multiple comparisons using familywise error (FWE) correction. For whole-brain analyses, we made inferences at cluster level (FWE: *p* < 0.05, corresponding to a cluster size of >140 on the basis of intensity threshold, *p* < 0.001). For VOI analyses, we made inferences at voxel-level (FWE corrected, *p* < 0.05; Worsley et al., 1996; Friston, 1997). Anatomical inference is drawn by superimposing SPM showing significant signal changes on structural images of participants. For anatomical accuracy, we report only activation peaks in gray matter. Anatomical landmarks were identified using the atlas of Duvernoy et al. (1991). Brodmann areas (BAs) were assigned by superimposing significant SPM on the SPM anatomy toolbox (Eickhoff et al., 2005) and MRIcron template (http://www.mccauslandcenter.sc.edu/mricro/mricron/).

### Connectivity analyses

The aim of the following analysis was to test whether inter-regional coupling of the aPFC (see Results) with the amygdala and other brain regions during the congruency effect was different between the groups and modulated by testosterone. To test for these effects, we used the psychophysiological interactions (PPIs) method (Friston et al., 1997). More specifically, we tested for significant differences between the regression coefficients of each voxel over the right aPFC during the affect-incongruent versus the affect-congruent conditions. To select voxels to be included in the VOI, we used the following anatomical constraints (Stephan et al., 2010): for each participant, selected voxels fell within a sphere with a radius of 4 mm around the peak voxel corresponding to the activated cluster of the congruency effect over both groups (coordinates: *x*, 30; *y*, 58; *z*, 14; see Results). Participant specific contrast images were generated describing the PPI between the time courses of the right aPFC VOI and affect-incongruent versus affect-congruent conditions. Group differences and testosterone modulations on task-related coupling between the aPFC and other regions were then assessed using a multiple regression design on participant-specific contrast images with their corresponding testosterone (log-transformed, standardized) and STAI state (standardized) levels as subject- and group-specific regressors. In addition to whole-brain analyses, we assessed significant voxel-level effects (FWE corrected for multiple comparisons, *p* < 0.05) within the amygdala, defined on the Automated Anatomical Labeling atlas (Tzourio-Mazoyer et al., 2002) using the WFU PickAtlas tool (Maldjian et al., 2003).

## Results

### Behavioral results

Fifteen psychopathic criminals (PPs; PCL-R score of ≥26, according to European standards (Rasmussen et al., 1999; Hare, 2003; Hildebrand et al., 2004) and 19 HCs (for demographics, see Table 1) were included in the analyses. Participants performed the task accurately and consistently (error rates: PPs, 7.9%; HCs, 7.3%; omissions: PPs, 1.6%; HCs, 1.5%; undefined responses: PPs, 0.9%; HCs, 0.3%; Table 2).

**Table 2:** RTs and error rates for each group and factor of the AA task

	Psychopathic offenders		HCs	
	Approach	Avoid	Approach	Avoid
Errors (%)				
Happy	3.2 (0.9)	8.9 (1.8)	2.4 (0.8)	7.7 (1.1)
Neutral	6.1 (1.3)	5.8 (1.1)	7.1 (1.4)	5.2 (1.0)
Angry	10.1 (2.2)	13.1 (2.1)	9.6 (1.8)	11.6 (1.8)
RT (ms)				
Happy	554 (25)	625 (35)	553 (23)	603 (25)
Neutral	666 (28)	687 (31)	639 (21)	668 (24)
Angry	630 (25)	665 (33)	620 (24)	630 (23)

Values are presented as the mean (SE).

**Figure 2.**
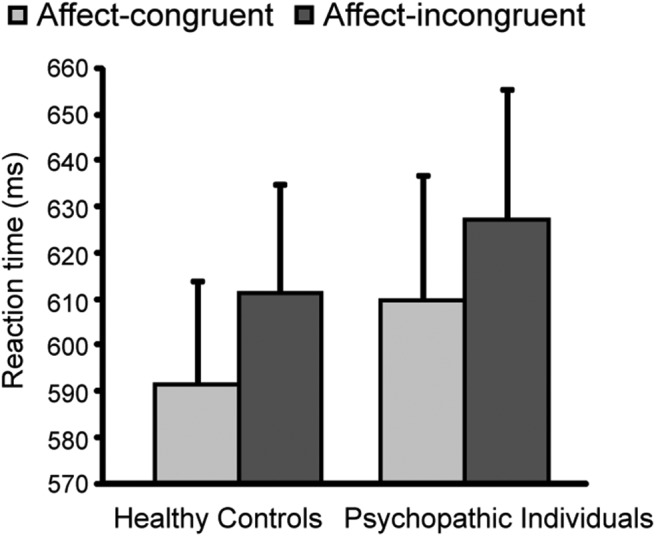
Behavioral results. Mean RTs (±SEM) for the affect-congruent and affect-incongruent conditions of the AA task for the healthy control subjects and psychopathic offenders. The groups were significantly slower to provide affect-incongruent responses (approach–angry; avoid–happy) than affect-congruent responses (approach–happy; avoid–angry), with no significant group differences.

A significant movement × valence interaction for the RTs indicated that, over groups, participants responded more slowly during affect-incongruent (approach–angry, avoid–happy) than during affect-congruent trials (approach–happy, avoid–angry; *F*_(1,29)_ = 10.4, *p* = 0.003; Fig. 2). This congruency effect replicates the behavioral results from previous fMRI studies (Roelofs et al., 2009; Volman et al., 2011b, 2013). Furthermore, there were main effects of movement (*F*_(1,29)_ = 26.3, *p* < 0.001) and valence (*F*_(1,29)_ = 28.7, *p* < 0.001), reflecting the slowing of avoidance movements and responses to angry faces in general (Table 2). There were no significant effects involving group, including no main effect (*p* > 0.3). The congruency effect correlated positively (without corrections for multiple comparisons) with the PCL-R total score (*p* = 0.048, *R* = 0.517, respectively). Excluding anxiety from the analyses did not affect the outcomes. Moreover, when including the neutral conditions in the analyses, the movement × valence (happy, neutral, angry) interaction for RTs remained significant (*F*_(1,28)_ = 5.5, *p* = 0.010), showing that neutral approach–avoidance effects are intermediary compared with happy and angry (Table 2).

For the error rates, the three-way ANCOVArm showed main effects of movement (*F*_(1,29)_ = 27.5, *p* < 0.001), valence (*F*_(1,29)_ = 25.9, *p* < 0.001), and testosterone (*F*_(1,29)_ = 4.6, *p* = 0.040), and a valence × testosterone interaction (*F*_(1,29)_ = 4.3, *p* = 0.047). There were no other significant effects for the error rates (*p* > 0.15).

Endogenous testosterone levels [median (SD): PPs, 101 pg/ml (70 pg/ml); HCs, 90 pg/ml (46 pg/ml)] and state anxiety levels [STAI mean (SD): PPs, 32 (8); HCs, 32 (5)] did not differ between groups (*p* > 0.4), and showed no correlations with psychopathy (PCL-R) scores or with each other (*p* > 0.1).

### fMRI results

#### Multiple regression analyses

To assess the two main questions of this study, we isolated cerebral structures showing stronger responses during affect-incongruent than affect-congruent trials (congruency effect), and cerebral structures in which the congruency effect was modulated by testosterone levels.

The results showed a significant congruency effect across groups in the aPFC [ROI analysis: MNI coordinates (*x*, *y*, *z*): (30, 58, 14) and (−30 58 10); *p*_FWE_ = 0.001 and 0.036; *t* = 4.46 and 3.43; for further details, see Table 3]. As expected, this effect was driven by the healthy control group, and it was significantly weaker in the psychopathic offenders [*p*_FWE_ = 0.001 and 0.040; *t* = 4.58 and 3.40, on the congruency effect in healthy control subjects masked implicitly by group (HC > PP) × congruency interaction]. The implicit masking demonstrates that the group × congruency interaction is also significant at *p*_uncorrected_ < 0.05 within the significant voxels corrected for multiple comparisons on the HC congruency effect. The psychopathy group showed no significant congruency effect in this region (*p*_FWE_ > 0.3). There was also a significant congruency effect across groups in the right superior parietal lobule (whole-brain analysis); this effect was driven mainly by the psychopathy group (Table 3).

**Table 3:** Clusters showing significantly larger activity for the affect-incongruent vs the affect-congruent conditions (emotion-control effect)

Anatomical region	Putative BA	Side	*x*	*y*	*z*	Voxels (*n*)	*p* value	*t* value
**Whole-brain effects**								
*Congruency effect over groups*								
Cuneus	18	R/L	−16	−96	24	634	<0.001	4.85
SPL/Superior occipital gyrus	7/19	L	−30	−76	34	254	0.004	4.37
IFG	45/47	L	−52	22	−10	223	0.007	4.37
Cuneus	18	R	18	−98	16	190	0.016	4.52
*Congruency effect for psychopathy group*								
SPL/superior occipital gyrus	7/19	R/L	8	−82	38	925	<0.001	4.98
Angular gyrus	39/19	L	−30	−72	34	337	0.001	4.35
Superior temporal gyrus	42	L	−32	−32	6	214	0.009	4.51
SPL	7	R	28	−74	46	158	0.034	4.58
Cerebellum		L	−24	−66	−38	146	0.046	4.63
*Negative testosterone modulation of group (psychopathic offenders > healthy control subjects) × congruency interaction*								
aPFC	10	R	30	58	12	391	<0.001	5.10
Supramarginal gyrus	40	R	54	−42	54	325	0.001	4.66
Caudate nucleus		R	10	10	2	273	0.002	4.69
Putamen/Insula		L	−34	6	−10	176	0.022	5.22
Cerebellum		R	18	−76	−38	188	0.016	5.09
*Negative testosterone modulation of Congruency effect in psychopathy*								
Supramarginal gyrus	40	R	52	−40	54	657	<0.001	5.32
Precentral/superior frontal gyrus	6	R/L	6	22	66	471	<0.001	5.65
Caudate nucleus		R	6	6	4	228	0.006	4.28
**VOI on bilateral aPFC**								
*Congruency effect over groups*	10	R	30	58	14	31	0.001	4.46
	10	L	−30	58	10	5	0.036	3.43
*Congruency effect in healthy control subjects*	10	R	32	58	14	12	0.001	4.58
	10	L	−34	52	4	2	0.040	3.40
*Negative testosterone modulation of group (psychopathic offenders > healthy control subjects) × congruency interaction*	10	R	30	58	12	145	<0.001	5.10
	10	L	−24	56	6	15	0.010	3.87
*Negative testosterone modulation of congruency effect in psychopathy*	10	R	32	56	10	77	0.002	4.34
	10	L	−30	58	8	17	0.015	3.74

Coordinates are defined in MNI (*x*, *y*, *z*) space. The *p* values represent the FWE cluster-level corrected values for the whole-brain analyses and FWE voxel-level corrected values for the VOI analyses. IFG, Inferior frontal gyrus; L, left; R, right; SPL, superior parietal lobule.

**Figure 3.**
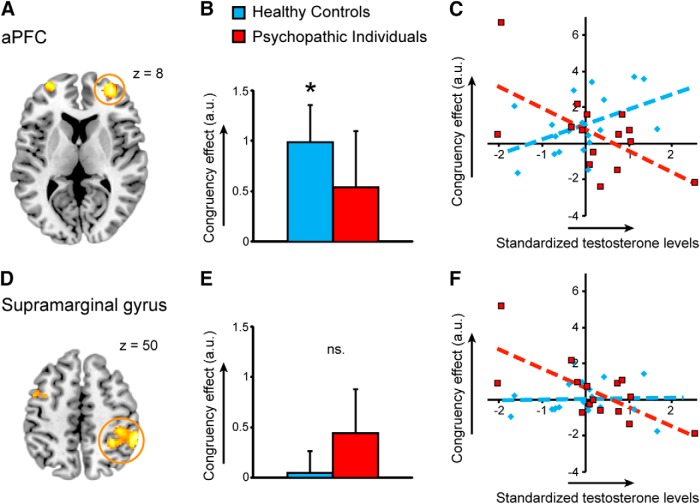
Testosterone modulations of the cerebral congruency effect in psychopathic offenders and healthy control subjects. ***A***, ***D***, Brain image showing testosterone-modulated congruency effects (affect-incongruent−affect-congruent) in the psychopathic offenders in the bilateral aPFC (***A***) and right supramarginal gyrus (***D***). ***B***, ***E***, Bar graphs showing the mean activation (±SEM) of the active voxels within the yellow circles per group. *p_FWE_ < 0.05. ns, Not significant. ***C***, ***F***, Scatterplots showing the correlation of the mean activation of active voxels within the yellow circles with testosterone (log-transformed and standardized) for the healthy control group and the psychopathy group. The ROI activations are presented at *p* < 0.05, uncorrected for visualization purposes. There are no outliers [Mahalanobis distances *D* ^2^*_i_* < 4.2 (cutoff at *p* < 0.05; *D* = 7.74); Barnett and Lewis, 1978; Stevens, 1996]. Healthy control subjects show an increased aPFC activity for the congruency effect and no modulation by testosterone, while in psychopathic offenders endogenous testosterone levels modulate the activity of the aPFC and right supramarginal gyrus.

Critically, testosterone modulated the congruency effect in the aPFC differently in psychopathic offenders and healthy control subjects (whole-brain analysis on testosterone × group × congruency: MINI coordinates (*x*, *y*, *z*): (30, 58, 12); *p*_FWE_ < 0.001; *t* = 5.10; for all details, see Table 3). *Post hoc* analyses revealed that, in the psychopathy group, congruency effects decreased as testosterone levels increased [MNI coordinates (*x*, *y*, *z*): (32, 56, 10) and (−30, 58, 8); *p*_FWE_ = 0.002 and 0.015; *t* = 4.34 and 3.74]. The modulatory effect of testosterone on congruency was absent in the healthy control subjects (*p*_FWE_ ≥ 0.05; Fig. 3*A–C*). The whole-brain analysis also showed an effect in the right caudate nucleus and right inferior supramarginal gyrus, driven by reduced congruency effects as a function of testosterone in the psychopathy group (Fig. 3*D–F*; Table 3).

#### Effective connectivity analyses

**Figure 4.**
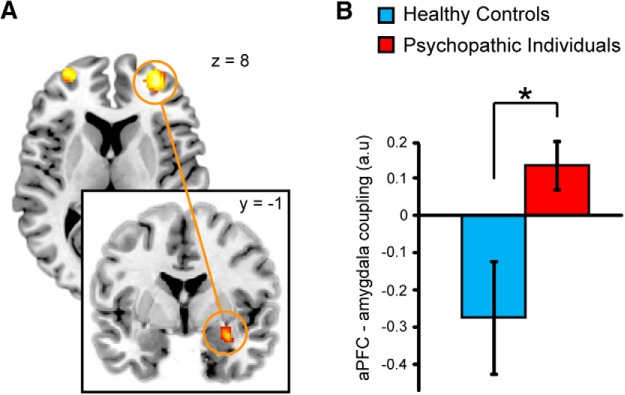
Group difference on congruency-related aPFC–amygdala connectivity. ***A***, Brain images illustrating the congruency-related modulation of connectivity between the right aPFC (yellow circle, axial slice) and the right amygdala (coronal slice) for the congruency contrast. The activations are presented at *p* < 0.05, uncorrected for visualization purposes. ***B***, Bar graph visualizing the strength of the congruency-specific change (±SEM) in aPFC–amygdala connectivity for the healthy control subjects and psychopathic offenders. There is a significant negative aPFC–amygdala coupling in the healthy control subjects, which is not present in the psychopathic offenders.

Given the relevance of aPFC–amygdala connectivity for implementing emotional control as evoked by the AA task (Volman et al., 2011a, 2013), we assessed whether psychopathy also resulted in altered connectivity along that neural pathway. Connectivity analyses using the right aPFC [4-mm-radius sphere; central voxel from main analysis (MNI coordinates: *x*, 30; *y*, 58; *z*, 14)] as the seed region on the congruency effect indicated a significant group difference (PP > HC) with the right amygdala (Fig. 4*A*,*B*; ROI analysis; extent, 3 voxels; *t* = 3.82; *p*_FWE_ = 0.027; MNI coordinates of local maxima: *x*, 32; *y*, 0; *z*, −16). When testing effects for both groups separately, healthy control subjects showed a significant negative coupling between the right aPFC and amygdala (ROI analysis; extent: 3 voxels, *t* = 3.70; *p*_FWE_ = 0.036; MNI coordinates of local maxima: *x*, 32; *y*, 0; *z*, −16), while psychopathic offenders showed no differential connectivity effect. *Post hoc* testing on right amygdala voxels showing the group interaction (threshold, *p* < 0.05 FWE) indicated a significant positive correlation with testosterone over both groups (ROI analysis; extent, 1 voxel; *t* = 2.29; *p*_FWE_ = 0.029; MNI coordinates of local maxima: *x*, 32; *y*, 2; *z*, −16). There was no correlation between aPFC–amygdala connectivity and the PCL-R scores (*p* > 0.2).

## Discussion

This study indicates that psychopathic offenders show reduced aPFC activity as well as less aPFC–amygdala connectivity during the control of emotional behavior. Emotional control was measured by comparing affect-incongruent and affect-congruent approach–avoidance responses to emotional faces (congruency effect on the AA task; Roelofs et al. 2009). When healthy control subjects exerted emotional control, reaction times, aPFC activity, and aPFC–amygdala anticorrelations increased, confirming previous observations (Volman et al., 2011b, 2013). In contrast, psychopathic offenders did not show this typical control-related pattern of aPFC activity and connectivity. In addition, these effects were significantly modulated by endogenous testosterone. Namely, psychopathic individuals with relatively lower testosterone levels showed a neural activity and connectivity pattern that resembled the findings in healthy control subjects, while this pattern was absent in those with higher testosterone levels. This indicates that especially psychopathic individuals with high testosterone levels have less prefrontal regulation of amygdala-driven emotional actions when the control of emotional behavior is required.

### Emotional control in psychopathy

Imaging studies have illustrated an association between psychopathy and altered processing of fear, including altered amygdala responses (Blair, 2010; Moul et al., 2012; Decety et al., 2015), attentional deficits for peripheral stimuli (Baskin-Sommers et al., 2011), and moral/empathic insensitivity (Decety et al., 2013; Marsh and Cardinale, 2012). However, psychopathic offenders also show clear impulsivity problems (Hare, 2003), for example, when control is required during emotionally provoking situations. To address this relatively unexplored but crucial component of criminal psychopathy, we used a paradigm requiring rule-driven control of emotional actions. With this paradigm, it was possible to move beyond simple motor inhibition and to target the flexible control of emotionally driven action tendencies.

First, the aPFC (also called BA 10) was less active in psychopathic offenders as a function of testosterone. The aPFC is a region crucial for the control of social emotional behavior. When aPFC functioning is temporarily disrupted, participants have increased difficulty in overriding emotional tendencies with rule-driven behavior (Volman et al., 2011a). Moreover, the aPFC seems especially important for integrating and coordinating multiple cognitive processes to facilitate response selection (Ramnani and Owen, 2004; Haggard, 2008). For example, transcranial magnetic stimulation-induced reduction of aPFC functioning during the control of emotional behavior decreased activity in brain areas associated with rule selection (posterior parietal cortex), while both amygdala activity and automatic action tendencies increased (Volman et al., 2011a). The current study indicates that psychopathic individuals with especially high testosterone levels recruited the aPFC less when the control of emotional responses was needed. This finding suggests that they have reduced coordination of rule-based behavior with emotional information.

Second, connectivity between the aPFC and amygdala also differed significantly between groups. Healthy control subjects showed a negative aPFC–amygdala coupling during the control of social emotional behavior, whereas psychopathic individuals showed no significant coupling between these regions. Evidence of anatomical connectivity alterations between these regions in psychopathic individuals and the relation of that tract to social emotional behavior modifications support these findings (Von Der Heide et al., 2013). Although these results cannot resolve the direction of these connectivity effects, a previous study (Volman et al., 2013) using this paradigm showed an effective connectivity modulation of emotional control on the connection from aPFC to amygdala. Also, animal studies (Quirk and Gehlert, 2003) suggest strong prefrontal inhibitory connections that control automatic amygdala responses. The absence of this aPFC–amygdala coupling in psychopathic offenders suggests that in this group the aPFC has a reduced ability to inhibit amygdala-driven responses. This study used subtle emotional provocations, but stronger emotional events result in stronger amygdala responses, increasing the bias for automatic emotional behavior (Quirk and Gehlert, 2003). A lack of prefrontal control likely reduces the ability to inhibit these biases and lead to an increased expression of automatic emotional actions even when they are not beneficial (Quirk and Gehlert, 2003; Volman et al., 2011a).

Testosterone administration studies also illustrated a decoupling between the prefrontal cortex and the amygdala, suggesting that testosterone reduces the communication between the PFC and amygdala (Eisenegger et al., 2011; Van Wingen et al., 2011; Bos et al., 2012) and, within the AA task, reduces top-down control. The association between testosterone levels and enhanced social aggression and dominance seeking, and reduced impulse control in the general population (Van Wingen et al., 2011; Montoya et al., 2012; Carré et al., 2013) supports the relevance of testosterone in this process. Even amygdala responses to angry faces have recently been found to be enhanced after testosterone administration and in psychopathic individuals (Van Wingen et al., 2011; Carré et al., 2013; Radke et al., 2015). There is a clear association between testosterone and aggression after provocation, which has been related to reduced activity in the orbital frontal cortex, a region just ventral of the aPFC (Mehta and Beer, 2010). Interestingly, psychopathic offenders with lower testosterone levels displayed a pattern similar to that in healthy control subjects, while the psychopathic individuals with high testosterone levels showed less aPFC activity and aPFC–amygdala coupling. This could provide a potential vulnerability factor explaining the difference between the goal-directed “successful” psychopath and the “unsuccessful” psychopath with reduced impulse control (Gao and Raine, 2010; Anderson and Kiehl, 2012). We hypothesize that especially psychopathic individuals with high testosterone levels fail to inhibit amygdala-driven action tendencies using the aPFC during the control of emotional behavior.

Endogenous testosterone levels also modulated control-related activity in the supramarginal gyrus and caudate nucleus of the psychopathy group. The supramarginal gyrus was previously found to be involved during emotional control on the AA task in a healthy student sample (Volman et al., 2011b). Previous work indicated that it plays an important role in action organization (Jubault et al., 2007), and that psychopathic individuals show reduced supramarginal gyrus activity compared with control subjects when reasoning about other people’s emotional state (Sommer et al., 2010). The current findings, emphasizing the role of supramarginal gyrus during emotional control in psychopathic offenders with low testosterone levels, could indicate the facilitation of action preparation in trials with affect-incongruent stimulus–response mapping. The caudate nucleus is important for incorporating predicted action outcome, when selecting the most beneficial behavioral goal (Grahn et al., 2008), and has previously found to be larger in psychopathy (Glenn et al., 2010). In light of these findings, our results suggest that psychopathic offenders with low endogenous testosterone levels, as opposed to those with high testosterone levels, have more interference of automatic action tendencies and outcomes associated with the facial emotions (e.g., approach–happy) that are opposite to the required actions during affect-incongruent trials (Grahn et al., 2008).

### Interpretational issues

Individuals with psychopathy have been suggested to have difficulty recognizing emotional expressions. However, this impairment seems quite specific to fear, rather than the emotional expressions used here (anger and happiness; Marsh and Blair, 2008; Von Borries et al., 2012). Furthermore, the groups assessed in this study made comparable numbers of errors, suggesting that psychopathic offenders had no special difficulty in recognizing the clear emotional expressions used in this study.

This study used a relatively subtle manipulation to target the emotional control system. The rationale of this choice was to detect neural vulnerability markers without affecting behavioral performance. Psychopathic offenders performing a more salient behavioral version of the AA task showed reduced avoidance of angry faces (Von Borries et al., 2012). In this study, angry faces evoked numerically similar behavioral effects (Table 2) and, additionally, aPFC effects (*post hoc* inspection of extracted parameters). Although these observations could be interpreted as a sign that psychopathic offenders have a tendency to approach angry faces, those observations were not statistically significant between groups [behavioral and aPFC group effects on angry faces: *p* > 0.2; *p*_FWE_ = 0.271; *z* = 2.54, on the angry–congruency effect in healthy control subjects masked implicitly by group (HC > PP) × angry–congruency interaction]. Future investigation is needed to directly test whether more provocative paradigms induce specific effects for angry faces. A previous study (Volman et al., 2013) using this fMRI task in participants with genetic susceptibility for developing aggressive disorders, also found no group-specific behavioral effects. That study suggested that alterations of the aPFC–amygdala pathway might reflect a vulnerability factor for psychopathologies.

Previously, endogenous testosterone modulated the aPFC and aPFC–amygdala coupling in a sample of healthy students (Volman et al., 2011b). In that study, a different demographic group of healthy control subjects similarly showed a testosterone modulation of aPFC–amygdala coupling, but no testosterone modulation of aPFC activity. This difference in the strength of testosterone-modulatory effects might be related to between-group differences in age (mean healthy control subjects, 41; mean students, 22; Peper et al., 2011), educational level (staff of forensic psychiatric institute vs university students), or general anxiety [STAI trait, lower in healthy control subjects of the current study; mean (SD): 29 (4.4) and 34 (6.9), respectively; *t*_(37)_ = −2.605; *p* = 0.014]. A limitation of this study is the modest sample size. Our focus to exclude moderating factors of comorbid disorders (except antisocial personality disorder) and recent drug use has the advantage that the sample is relatively homogeneous, but future studies using larger samples are needed for replication and to define subsamples.

### Conclusion

Psychopathic offenders showed reduced aPFC activity and aPFC–amygdala connectivity during control of emotional actions, suggesting a decreased coordination of emotional information during rule-driven behavior. Moreover, endogenous testosterone modulated the involvement of these neural mechanisms. Psychopathic offenders with high testosterone levels showed less involvement of the aPFC, aPFC–amygdala connectivity, supramarginal gyrus, and caudate nucleus, whereas psychopathic individuals with low testosterone levels recruited the aPFC in a fashion similar to that of healthy control subjects. These findings suggest that a lack of prefrontal control during emotional actions may explain enhanced impulsivity in psychopathic offenders during emotionally provoking situations. They outline a neuroendocrine model underlying impulsive emotional behavior in psychopathy and support the relevance of assessing a potential imbalance in testosterone function to guide treatment. It remains to be seen whether these neuroendocrine alterations of emotional control are also present in highly impulsive or antisocial individuals.
